# Attention Detection by Heartbeat and Respiratory Features from Radio-Frequency Sensor

**DOI:** 10.3390/s22208047

**Published:** 2022-10-21

**Authors:** Pragya Sharma, Zijing Zhang, Thomas B. Conroy, Xiaonan Hui, Edwin C. Kan

**Affiliations:** School of Electrical and Computer Engineering, Cornell University, Ithaca, NY 14850, USA

**Keywords:** attention detection, radio frequency, vigilance, vital signs, wearable sensor

## Abstract

This work presents a study on users’ attention detection with reference to a relaxed inattentive state using an over-the-clothes radio-frequency (RF) sensor. This sensor couples strongly to the internal heart, lung, and diaphragm motion based on the RF near-field coherent sensing principle, without requiring a tension chest belt or skin-contact electrocardiogram. We use cardiac and respiratory features to distinguish attention-engaging vigilance tasks from a relaxed, inattentive baseline state. We demonstrate high-quality vitals from the RF sensor compared to the reference electrocardiogram and respiratory tension belts, as well as similar performance for attention detection, while improving user comfort. Furthermore, we observed a higher vigilance-attention detection accuracy using respiratory features rather than heartbeat features. A high influence of the user’s baseline emotional and arousal levels on the learning model was noted; thus, individual models with personalized prediction were designed for the 20 participants, leading to an average accuracy of 83.2% over unseen test data with a high sensitivity and specificity of 85.0% and 79.8%, respectively

## 1. Introduction

Ambient intelligence and intelligent machine responses [1,2] have become increasingly important in recent years, and both require an estimate of human cognitive reactions. Attention detection is a subset of cognition assessment that can enable accident prevention by warning when the user starts slipping into an inattentive state. This is important for activities of daily living, including driving, as well as certain occupations, such as the military [3,4], medicine, aviation, etc. With increasing numbers of work-from-home jobs, such systems are even more important for individuals to monitor self-work fatigue and take recuperative measures.

When people perceive a vast amount of information, a subset processing is prioritized and extraneous irrelevant information filtered out, which is termed as attention [5]. It is a basic function that simultaneously controls focus, vigilance, and response [6]. Two broad attention types are endogenous and exogenous. The former is a top-down, goal-driven voluntary process with the conscious expectation of events, while exogenous attention is a bottom-up, sensory-driven, involuntary response. The attention time course can last for a short duration (a few milliseconds) to longer periods (a few seconds or minutes), termed as sustained attention or vigilance [7]. Some long-term tasks, commonly associated with workplaces, require vigilance, and may result in mental and physical fatigue.

The literature is abundant in the study of attention-related concepts, including alertness, fatigue, and engagement. Engagement is closely related to attentional involvement with a task, mainly detected using facial expressions [8,9]. Fatigue induced drowsiness impacts attention by decreasing the ability to suppress irrelevant information, leading to increased reaction times [10,11]. With more than 300,000 drowsy-driving crashes each year [12], numerous research works have made significant efforts towards driver fatigue and sleepiness detection [13], primarily using change in the blink rate, percentage of eye closure (PERCLOS), facial expression, and voice features, all of which are typical indicators of arousal level [14]. Thus, camera-based technologies including video, audio, and infrared (IR) illuminators, play a major role in observing the psychological condition [15]. This setup is feasible in confined spaces, with a fixed relative user–sensor position; however, it may be impacted by environmental factors including ambient noise, poor illumination, or even sunglasses [16,17].

Compared with audio–visual responses for psychophysiological monitoring, involuntary reactions, such as variation in brain waves, heartbeat, respiratory patterns, skin conductance (SC), and skin temperature (ST), can be more objectively measured. In general, the electroencephalogram (EEG) has been extensively used to understand cognitive conditions including attentiveness, with Liu et al. achieving 76.8% accuracy [18]. While EEG frequency bands contain important information to detect sustained or selective attention [19], degree of inattentiveness is more difficult to identify [18]. Electrooculography (EOG) can measure long blink durations and slow eye movements, which are indicators of sleepiness and reduced attention [20]. Furthermore, SC, measured from electrodes on fingers or hands has also been used for mental workload assessment [21]. Heart rate (HR) and heart rate variability (HRV) allows study of fluctuations in the sympathetic and parasympathetic nervous systems (SNS, PNS, respectively) and, thus, have been used extensively with good performance [22]. The changes in HRV, blood pressure, and palm ST have been utilized for drowsiness detection [23]. Additionally, the ECG reliably captures high-quality heartbeat signals, but all these electrode-based sensors face similar setup issues, in that they have a skin-contact requirement and are uncomfortable, despite advances in dry electrodes and wireless setup. A photoplethysmogram (PPG) from a wrist-band is a more comfortable, long-term alternative to ECG; however, it has limited accuracy in comparison [24]. Respiration has been hypothesized to be impacted by attention and stress levels and, in turn, might impact these [25] due to unique dual autonomous and voluntary nature. Breathing may capture similar features as HRV, since they are linked by respiratory sinus arrhythmia (RSA) [26]. However, this has been studied less due to the uncomfortable monitoring with thorax and abdominal tension belts or a nasal cannula. Few works have studied its link to emotion [27,28] and attention [29]. The best performance can be achieved with a simultaneous multi-sensor unit, which may be inconvenient due to multiple-point skin contact electrodes and headband [30].

This work proposes a study of attention using a noninvasive radio-frequency (RF) sensing technology [31] that detects both respiration and heartbeat motion with high comfort and minimal distraction. Our near-field coherent sensing (NCS)-based RF sensor couples strongly to the near-field motion of heart, lungs, and diaphragm, clearly capturing every heartbeat, inspiration, and expiration duration with high resolution, without any impact of ambient motion [32]. In comparison, the existing RF sensing techniques measure reflection from the body surfaces [33], mainly consisting of respiration motion, which is 10× heartbeat surface motion [34]. The reflected signal is also easily interfered with due to ambient motion that needs to be suppressed by advanced hardware and signal processing techniques [35,36]. Naturally, the existing RF technique has limited use for a fixed home-based setting with one user, as it is difficult to decouple vitals and associate them with users without added directionality sensing.

We have evaluated our sensor and feature extraction for attention vs inattention classification on 20 healthy participants. Two RF sensors were worn at the thorax and abdomen levels to monitor heartbeat, thorax, and abdominal respiration during the inattentive and engaged-attentive states. A Mackworth clock task was used in the attention routine [37] to estimate vigilance, which mimicked scenarios where the user needed to be continuously attentive, such as in driving or guarding. Cardiac and respiratory features were extracted from the collected waveforms and fed into the machine learning (ML) model for classification. A questionnaire at the end of the study revealed varying baseline arousal and emotional states including calmness, drowsiness, and anxiety. The major contributions of this work include the following:

1. A touchless RF sensor that measures both cardiac and respiratory waveforms, with on-par attention detection performance as the reference chest tension belts and ECG together. The improved comfort and convenience can reduce the systematic bias and improve the applicability;
2. Both cardiac and respiratory variability features were employed to derive the attention status every 10 s by a learning model, which were more accurate than the individual cardiac and respiratory features;
3. The critical role of personal baseline training was examined.

Section 2 presents the RF sensor and experimental setup. The algorithm for feature extraction is discussed in Section 3. Results are presented in Section 4, followed by the discussion and conclusions.

## 2. Experimental Design

### 2.1. Sensor Setup

**Figure 1:**
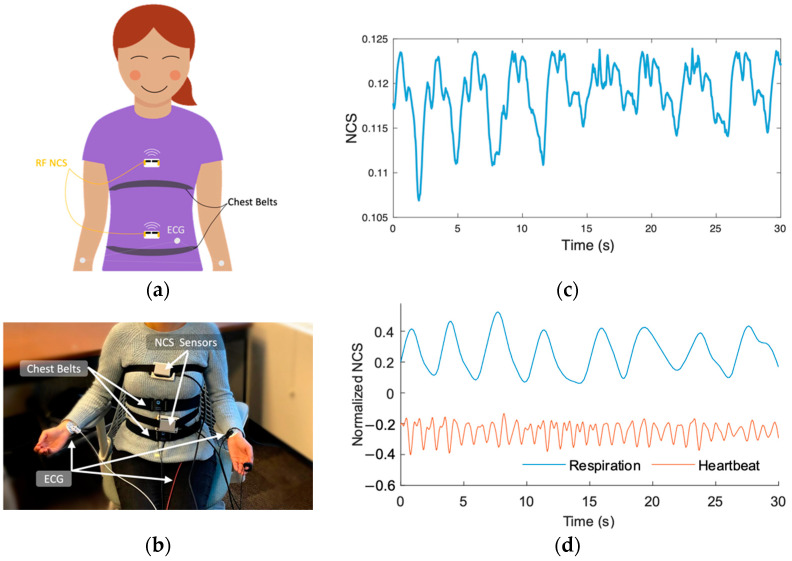
(**a**) Setup showing NCS RF sensors near the heart and diaphragm, and reference thorax and abdomen chest belts and ECG. (**b**) A subject wearing the setup, with wired NCS sensors. Newer version enables Bluetooth data transfer. (**c**) A 30 s segment showing raw respiration and heartbeat modulation on NCS signal. (**d**) Filtered and normalized heartbeat and respiration extracted from the NCS thorax and abdomen sensors, respectively.

The hardware setup included two over-the-clothes RF NCS sensors placed at the thorax and abdomen levels on the midline, as shown in Figure 1a,b. The wired RF sensors were held in place by belts, with no tension requirement. The newer lightweight Bluetooth-enabled design allows for a more comfortable alternate placement [38]. The heartbeat signal was generally stronger in the thorax sensor, as it was placed closer to the heart. The abdomen sensor had a stronger lung and diaphragm motion. Figure 1d shows the typical heartbeat and respiration waveforms extracted from the NCS sensors. The sensor prototypes are implemented using a software-defined radio (SDR) transceiver by Ettus Research [39], operating at 1.82 GHz and 1.9 GHz with <−10 dBm power. A detailed description was presented in our previous work [32]. The reference sensor setup included a three-electrode ECG, and thorax and abdomen chest belts by BIOPAC [40]. Notice that ECG electrodes required conductive gel pads with bare skin touching, and that the chest belts needed reasonable tension to capture the full respiratory motion.

### 2.2. Protocol

**Figure 2:**
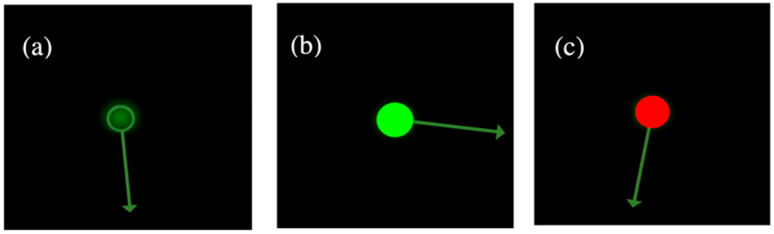
Different clock hand status during the attention task. (**a**) Normal clock rotation; (**b**) abnormal clock rotation, after correctly detection by user; (**c**) incorrect user response due to missed abnormal rotation or spacebar press for a normal clock rotation.

The protocol included two routines in seated posture, namely relaxed inattentiveness (R) and vigilance-attention (A). In the former, participants were asked to relax with eyes closed for 5 min, maintaining a state of inattentiveness. The next routine involved a stimulus-driven attention task, demanding sustained vigilance during a modified Mackworth clock game [37]. A rotating clock hand was shown on the computer screen and participants were expected to respond to larger clock hand jumps by pressing the spacebar. A maximum reaction time (RT) of 1 s was allowed. The entire 6.5 min routine included some instructions and a trial run of 1 min, followed by same vigilance task for 5 min, and finally 30 s looking at the screen for potential future instructions. As participants were expected to be attentive during the entire 6.5 min routine, the entire duration is considered as an attention routine. The routine was designed using PsyToolkit software [41] which showed a clock hand rotating by a fixed step of 3.6°/s. The probability of a longer rotation of 5.4° at each step was set as 0.1. An indicator at the center of the clock gave instantaneous feedback of incorrect, missed, and correct responses by red, red, and green lights, respectively. Figure 2 shows different possible clock states during the task.

The experimental study was approved by the Cornell Institutional Review Board (IRB) and conducted with the written consent of the participants. Data collection was performed on 20 healthy participants including 12 female and 8 male subjects. The age range was 18–34 years, with BMI in the range 18–27 kg/m^2^. An end-of-study questionnaire noted participants’ feelings of stress, relaxation, calmness, anxiety, and alertness during both routines.

## 3. Data Processing and Feature Extraction

### 3.1. Sensor Data Preparation

Our setup collected timestamp synchronized NCS and BIOPAC data, along with the information of each clock hand step and keypress RT. The NCS respiration and heartbeat waveforms were modulated on the baseband RF amplitude and phase waveforms, and were extracted by filtering [42]. For respiration, low-frequency baseline variation was removed with an order-5 Butterworth filter and 3 dB cutoff frequency ($f_{3dB}$) of 0.05 Hz. A low-pass FIR Kaiser-window filter was used to suppress high-frequency heartbeat waveforms over 0.8 Hz. The resulting waveform was further processed by subtracting the mean of the first 60 s of data, followed by normalization using RMS of the same duration. Similarly, the heartbeat waveform was extracted by a third-order high-pass filter with 0.7 Hz $f_{3dB}$ and a similar low-pass filter with 1.9 Hz cut-off. These filters allow measurement of respiration rate (RR) and heart rate (HR) in the ranges of 6–40 and 45–115 breaths or beats per minute (BPM), respectively, well over the normal resting range. All vitals were down-sampled to a uniform sampling rate, $f_{s}$ = 100 Hz before feature extraction.

### 3.2. Dual-Point NCS Measurement

As discussed earlier, we had a two-sensor placement that measured heartbeat primarily from the thorax and respiration from both thorax and abdomen sensors. Furthermore, vital-sign modulations were observed in both amplitude and phase of the baseband RF signal. Thus, the NCS signal entropy, was high with four signal sources (thorax amplitude, thorax phase, abdomen amplitude, and abdomen phase) for respiration and heartbeat waveform extraction. This redundancy was particularly useful when external motion artifacts are present, which would not affect all four channels in a similar manner. A signal-to-noise ratio (SNR) estimate was defined to identify the signal with the best quality. Signal and noise powers were estimated from the periodogram of power spectral density after baseline removal ($f_{3dB}$ = 0.08 Hz). SNR was derived as follows:

(1) $$\mathrm{SNR}=10\cdot \log_{10}\frac{P_{f_{1}}}{P_{f_{2}}+P_{f_{3}}}\;,$$

where $P_{f1}$ indicates the power in the desired signal frequency band $f_{1}$, and $P_{f2}$ and $P_{f3}$ are the noise bands after filtering. For heartbeat, $f_{1}$ is [0.8, 2] Hz, $f_{2}$ is [0.05, 0.8) Hz, and $f_{3}$ is (2, 50] Hz, where $f_{s}$/2 = 50 Hz is half of the sampling frequency. For respiration, $f_{1}$ is [0.1, 0.7] Hz, $f_{2}$ is [0.05, 0.1) Hz, and $f_{3}$ is (0.7, 50] Hz. Here, we have not differentiated between intensity of thorax and abdomen breathing, and the waveform with the highest SNR was selected for feature extraction.

### 3.3. Feature Extraction

#### 3.3.1. Heart Inter-Beat Interval Detection

**Figure 3:**
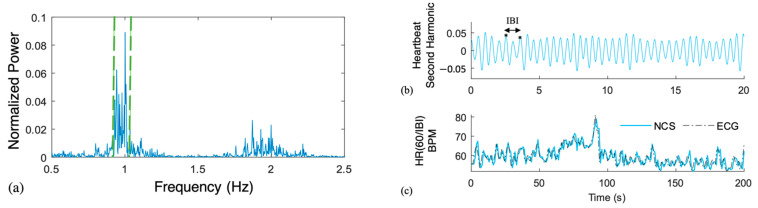
(**a**) Frequency spectrum of NCS thorax waveforms during the relaxation routine of 5 min showing variable HR in the range [0.93,1.04] Hz, indicated by the dashed green lines. Similarly, the second harmonic of heartbeat is also distributed around 2 Hz. (**b**) Second harmonic NCS heartbeat waveform, where IBI is taken as the time for two cycles. (**c**) Instantaneous HR (60/IBI) in BPM from NCS and ECG showing high correlation. The HR shows strong variation from 50–80 BPM in a resting state.

The HR is not stationary over time, and its variability contains valuable information about the SNS and PNS response [43]. Figure 3a shows the frequency domain characteristics of the NCS thorax waveform between [0.5, 2.5] Hz, visibly showing variable HR in the range of [55.8, 62.4] BPM. To accurately extract the inter-beat interval (IBI) from the smooth NCS signal, a weaker, but sharper second harmonic heartbeat component of the heartbeat was used [32] and IBI was measured as the time for two cycles, as denoted in Figure 3b. This process resulted in very accurate instantaneous HR estimation compared to the reference ECG as shown in Figure 3c. Note that ECG measures the electrical stimulation while NCS measures the actual heartbeat motion. The peak points in the waveform were extracted by a robust algorithm using the intersection of the moving average curve (MAC) [32].

#### 3.3.2. Respiration Waveform Extrema Detection

To investigate the correlation between heartbeat and respiration, we have designed statistical features representing respiration waveform variability (RWV) utilizing inspiration, expiration, and respiratory effort information. The peaks in respiration waveform were extracted by the same MAC algorithm. For respiration, maxima peaks represent the end of inspiration, termed as inspire-end points, $t_{e}$. The beginning of inspiration is represented by the inspire-begin point, $t_{b}$, which is more difficult to accurately capture using a minima-detection algorithm. We attribute this to the following reasons: (1) a longer exhalation breath pause leading to relatively flat waveform; (2) filter artifacts can change the true minimum, especially around pauses; (3) unfiltered small heartbeat or pulse motion can result in multiple local minima. Thus, a post-processing approach was employed to identify true $t_{b}$, as follows:

1. Find zero-crossing (ZC) points of the first derivative (${\mathrm{ZC}}_{1}$) and second derivative (${\mathrm{ZC}}_{2}$) of the respiration waveform $r\left(t\right)$ between consecutive inspire-end peaks, $t_{e}$ and $t_{e-1}$;
2. Select only positive slope points of ${\mathrm{ZC}}_{2}$ (${\mathrm{ZC}}_{2+}$), with the first-derivative close to 0 (${\mathrm{ZC}}_{2+}^{0}$);
3. Identify all such points b∈{${\mathrm{ZC}}_{1}$,${\;\mathrm{ZC}}_{2+}^{0}$} as possible minima if $r\left(t_{e}\right)-r\left(t_{b}\right)>0$, and $\left(\underset{b}{\max}r\left(t_{e}\right)-r\left(t_{b}\right)\right)/\left(\underset{b}{\min}r\left(t_{e}\right)-r\left(t_{b}\right)\right)<2$.
  - If all are minima, select the point closest to inspire-end: b  that gives $\underset{b}{\min}\left(t_{b}-t_{e}\right)$.
  - Otherwise, select the minimum point b that gives $\underset{b}{\min}\left|r\left(t_{b}\right)\right|.$

**Figure 4:**
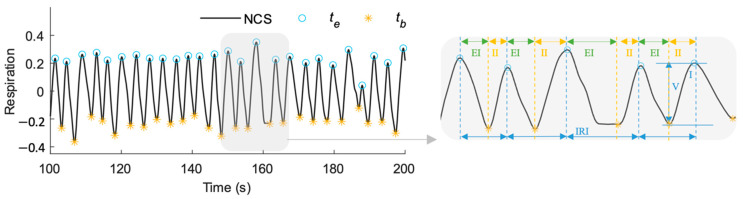
NCS respiration waveform showing different features. The maxima and minima represent inspire-end $\left(t_{e}\right)$ and inspire-begin $\left(t_{b}\right)$, respectively. IRI = $t_{e}-t_{e-1\;},$ II = $t_{e}-t_{b}$, EI = $t_{b}-t_{e-1},$ and IV = $r\left(t_{e}\right)-r\left(t_{b}\right)$. Accurate inspire-begin point is estimated for a difficult case with a pause after exhalation at t=162 s.

This results in an accurate $t_{b}$ corresponding to each $t_{e}$ to define the respiratory features, allowing independent study of inspiration and expiration variability which has not been explored in detail in earlier works [44]. Figure 4 shows the respiration waveform annotated with the detected $t_{b}\;\mathrm{and}\;t_{e}$ along with corresponding inter-respiration interval (IRI), inspiratory interval (II), expiratory interval (EI), and inspiratory volume (IV) estimations for individual breath cycles.

#### 3.3.3. Heartbeat and Respiratory Features

The detected heartbeat IBI and respiration IRI, II, EI, and IV parameters were used to calculate features over each windowed segment. Both R and A routines were divided into 90 s windows ($t_{win}$), or epochs, with a 10 s sliding interval ($t_{slide}$) over which ultra-short HRV [45] and RWV features were estimated. For HRV analysis, standard time and frequency-domain metrics were derived from NCS and ECG, as follows:

1. The mean(HR), mean(IBI), and std(IBI) are the mean and standard deviations of HR and IBI, after rejecting poor IBI values;
2. The pIBI50 is the ratio of successive IBI counts that differ by more than 50 ms to the total IBI count, closely related to PNS activity;
3. The LF, HF, and LF/HF are the power in low-frequency (LF:0.04–0.15 Hz), and high-frequency (HF: 0.15–0.4 Hz) indicating a balance between the SNS and PNS activity [22].

The RWV features included mean and standard deviation of IRI, II, EI, and IV, their first and second successive differences (SD_1_, SD_2_) and the ratio of EI/II that is known to be related to the stress level [46]. The RR was also estimated as a function of the mean(IRI) over 15 s. All 36 RWV and 7 heartbeat features are listed in Table 1. The RWV features were estimated from the highest SNR NCS respiration and reference chest belt waveforms. The nonlinear entropy-based features were found to be unreliable with high dependence on the sample size [47], and were not included.

**Figure 5:**
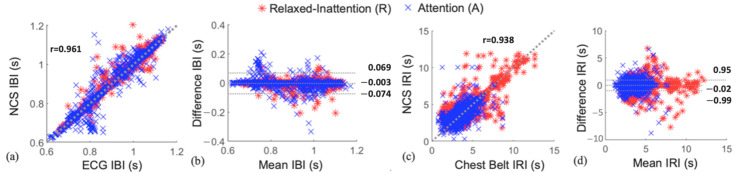
Comparison of NCS and reference IBI and IRI data from all participants during relaxed–inattention and attention routines. (**a**,**c**) The IBI and IRI scatter plots with denoted Pearson’s correlation coefficients; (**b**,**d**) The Bland–Altman plots showing the mean difference *m* at the center dotted line and the corresponding LoA.

In Figure 5a, we present the correlation plot between NCS and ECG IBI data, achieving a high Pearson’s correlation coefficient r = 0.961. The Bland–Altman plot in Figure 5b presents high agreement between the two measurements. The X axis is the average of the two data, and the Y axis is the difference. The middle-dotted line at −0.003 s shows a low mean (*m*) bias. The other lines show limits of agreement (LoA), within which 95% of the differences are expected, calculated as m±1.96⋅σ. Similarly, Figure 5c,d shows the scatter and Bland–Altman plots between NCS and the reference IRI. High correlation between NCS and the reference heartbeat and respiratory features confirms the accuracy and robustness of our system. Low *m* and narrow LoA indicate small, uncorrelated errors between NCS and reference estimates.

## 4. Results

**Figure 6:**
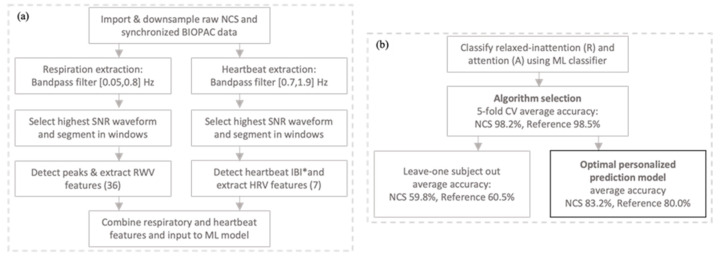
Flow charts representing our signal processing and ML system architecture. (**a**) Signal processing and feature extraction. * IBI is extracted from second harmonic heartbeat, requiring re-filtering of the highest SNR waveform around the dominant HR frequency; (**b**) ML model validation process.

In this section, we present accuracy statistics of NCS-based inattention vs attention detection. The current literature has mostly focused on HRV-based emotion and fatigue detection, due to high sensor reliability and higher comfort than the use of tension chest belts for the study duration. Here, this gap is closed with an additional performance comparison of respiratory and cardiac features. To further characterize users’ attentive state, correlation trends between probability of correct response (PoCR) and RT are presented for the short study duration. Furthermore, MATLAB toolboxes have been used for the following analysis. Figure 6 presents the system architecture flowchart, including (a) signal processing and feature extraction, and (b) the machine learning algorithm for attention detection.

### 4.1. Attention and Relaxed—Inattention Classification

For epoch-based analysis, the initial 10 s of R-routine data was rejected to allow participant to settle, reduce motion artifacts in data, and account for any potential delay in achieving the inattentive-relaxed state. For simplifying the current analysis, participants were suggested to stay stationary as much as possible, and early truncation was performed for two participants to reject poor motion artefact data. Thus, 290 s and 390 s were extracted from R and A routines from all participants, except for participants 3 and 12, with 370 s and 220 s A-routine data, respectively.

While we have a limited dataset of a small epoch size, this higher time resolution is advantageous, as a user’s sudden inattentiveness may be detrimental to the task. For attention vs relaxed–inattention classification, we tested two approaches, as follows: (1) leave one subject out, and (2) a personalized prediction model. A fixed algorithm was selected by 5-fold cross-validation (CV) for consistent comparison across both approaches. The kNN classifier achieved the best accuracy for binary attention vs relaxed inattention classification for each 90 s epoch, compared to SVM, QDA, and boosted and bagged tree algorithms, as shown in Table 2. The NCS and reference achieved similar accuracies of 98.2% and 98.5%, respectively, using all the described features in Section 3.3.

**Figure 7:**
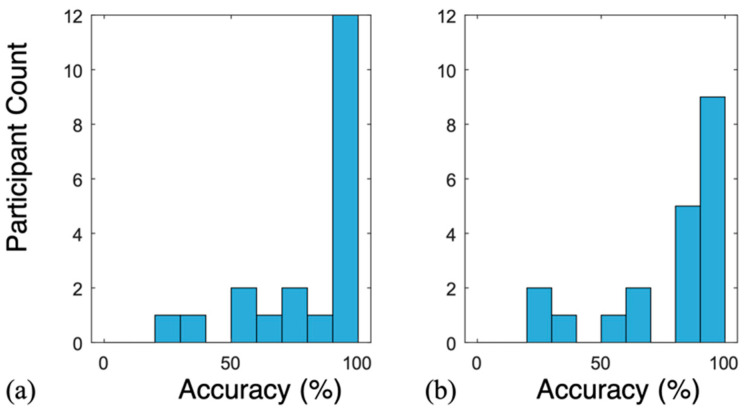
Test accuracy distribution across the participants using the personal calibration model by (**a**) NCS and (**b**) the reference.

Using the kNN model, the leave one subject out test resulted in an accuracy drop to 59.8% and 60.5% for NCS and the reference, respectively. This suggested a high personal baseline influence on the model, which has been consistent with other works in area [48]. In the second approach, personalized prediction models for each user were designed using a small subsection of data for training and remaining out-of-time data for testing. The beginning 180 s of data from both routines were used for training, with no time overlap between the training and test epochs. A smaller A-routine training duration was selected for Participant 12 to have one test epoch. A 50% holdout of training data was used for validation and tuning. Good test accuracies of 83.2% and 80.0% were achieved by NCS and the reference, respectively. Figure 7 shows the test accuracy distribution across all the participants. Detailed results for individual participants are in Table 3, showing 85.0% average sensitivity and 79.8% average specificity for classification by NCS.

We have attributed the test performance drop for some participants to variation in A and R levels over time. This is also consistent with the participant reports of varying attention levels over time. For example, Participant 7 reported, “*I maintained the same level of relaxation throughout the relaxation phase, but at the attention phase, I was more attentive at first, but slowly got less so towards the end.*” Some subjects felt increased drowsiness, according to Participant 14, “*I feel* [felt] *relaxed throughout the relaxation test phase, and slightly sleepy towards the end. During the attention test phase, I felt alert and slightly stressed as I got a couple wrong. Towards the end of the attention phase, I felt a little tired/hypnotized from looking at the small movements of the clock hand.*”

### 4.2. Cardiac and Respiratory Feature Comparison

**Figure 8:**
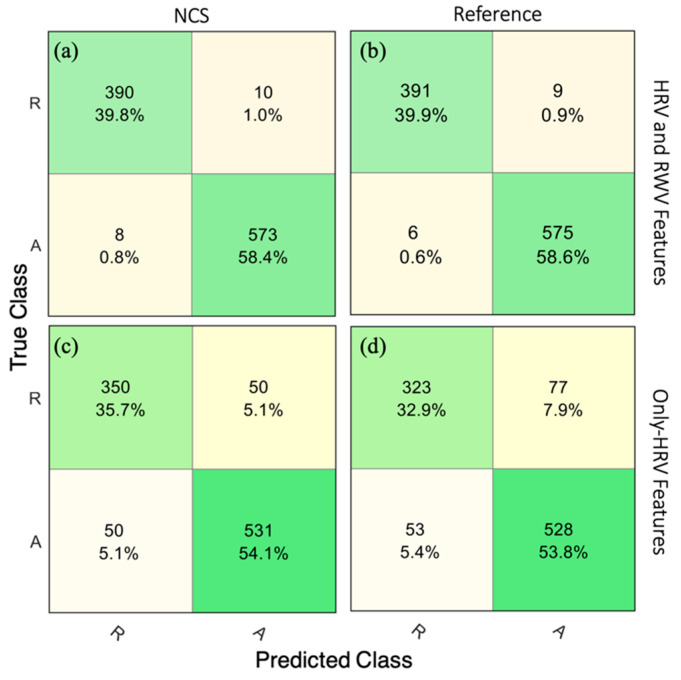
The 5-fold CV confusion matrices showing relaxed–inattention (R) and attention (A) classification by the kNN algorithm using NCS and reference HRV and RWV features in (**a**,**b**), and only HRV features in (**c**,**d**), respectively. The cells list number of epochs in each category and their overall percentage.

To understand the individual contribution of respiration and heartbeat features, classifier performance was tested with only one feature set at a time. This resulted in a higher average 5-fold CV accuracy of 97.7% from the RW model compared to 89.8% from the HRV model by NCS. A similar trend was observed with the reference ECG and chest belt sensors showing 96.9% and 86.7% accuracies using RWV and HRV features, respectively. Figure 8 shows confusion matrices for NCS with all features and only HRV features in Figure 8a,c, compared to the reference BIOPAC in Figure 8b,d. The NCS RWV-only model (97.7%) performed very close to the model using both RWV and HRV feature sets (98.2%), which suggests high attention-specific information in RWV features and overlap between RWV and HRV feature information. A potential reason for this is RSA coupling between respiration and heartbeat. The signal quality between RWV and HRV may play a role as well, as well as the small time duration for frequency-domain features.

### 4.3. Participant Response Characterization

As an extension of attention versus relaxed–inattention detection, we also investigated the response characteristics of participants during the attention routine to search for the correlations or trends among PoCR, RT, and HR. As the respiratory features have longer periods than the cardiac ones, we have selected HR for the study of short-term variation around each event.

**Figure 9:**
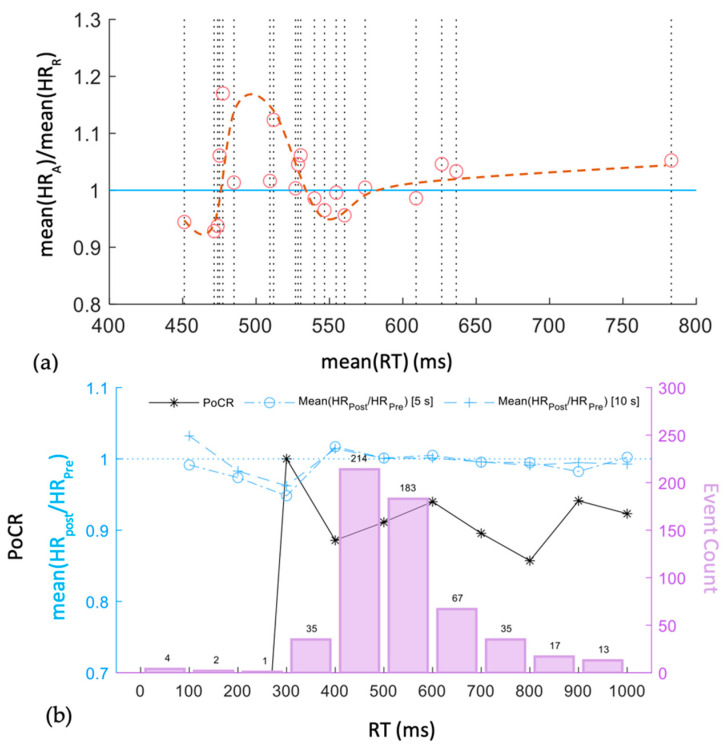
(**a**) Average HR change from the R to A routine as a function of RT; each circle represents a participant. (**b**) Trend analysis for each *event* across all participants. The PoCR is a function of RT for individual events. Trends of mean(${\mathrm{HR}}_{\mathrm{Post}}/{\mathrm{HR}}_{\mathrm{Pre}}$) for each event with two window sizes of 5 s and 10 s are shown in blue lines. The RT histogram across all events is shown with purple bars.

The first metric compares variation in average HR over both routines vs the RT of each participant. A ratio of average HR during the A and R routine ($\mathrm{mean}\left({\mathrm{HR}}_{A}\right)/\mathrm{mean}\left({\mathrm{HR}}_{R}\right)$) is plotted as a function of average RT ($\mathrm{mean}\left(\mathrm{RT}\right)$) for each participant in Figure 9a. Within the limited number of participants, we observed an increasing trend in $\mathrm{the}\;\mathrm{ratio}\;\mathrm{mean}\left({\mathrm{HR}}_{A}\right)/\mathrm{mean}\left({\mathrm{HR}}_{R}\right)>1$ as $\mathrm{mean}\left(\mathrm{RT}\right)$ approached 500 ms, and then gradually lowered to around 1 for the higher $\mathrm{mean}\left(\mathrm{RT}\right)$. An increase in HR could be associated with an increased stress or surprise. Thus, quick responses with mean(RT) < 475 ms and <1 ratio are likely associated with low stress, followed by an elevated stressed response, before the ratio dampened out to ~1 with high RT. Here, the $\mathrm{mean}\left(\mathrm{RT}\right)$ excluded the missed response cases with a fixed maximum RT.

The second metric takes correctness of response into account, in addition to RT. An *event* is described as any correct or incorrect user response towards the clock jump. The HR estimated in pre-event and post-event window sizes of 5 s and 10 s was used to calculate ${\mathrm{HR}}_{\mathrm{Post}}/{\mathrm{HR}}_{\mathrm{Pre}}$ for each event. The RT intervals associated with all events were distributed in 100 ms bins, and mean(${\mathrm{HR}}_{\mathrm{Post}}/{\mathrm{HR}}_{\mathrm{Pre}}$) metric was estimated along with PoCR in each RT bin, as shown in Figure 9b. Here, PoCR is defined as fraction of correct events out of total events. It is observed that mean(${\mathrm{HR}}_{\mathrm{Post}}/{\mathrm{HR}}_{\mathrm{Pre}}$) [5 s] < 1 with $\mathrm{RT}\in \left[200,\;400\right)\;$ ms, with similar trends for both window sizes. As RT increased to a range of [400, 600) ms, the PoCR oscillated around 0.9 with a slightly elevated mean(${\mathrm{HR}}_{\mathrm{Post}}/{\mathrm{HR}}_{\mathrm{Pre}}$), which stabilized for the higher RT. In other words, when a participant was expected to be in an attention state, the following scenarios could be expected:

1. A very quick reaction (RT≤200 ms) had a high probability to be incorrect;
2. A moderately fast response with $\mathrm{RT}\in \left[250,350\right)\;$ ms indicated a high PoCR and mean(${\mathrm{HR}}_{\mathrm{Post}}/{\mathrm{HR}}_{\mathrm{Pre}}$) ~ 1. This can be considered as the period when the user mastered the game with full attentiveness. However, (1) and (2) have small numbers of events (1 and 35, respectively), and the deduction can only be viewed as preliminary;
3. Most RTs were in the range of 400–600 ms. Interestingly, RT > 400 ms was associated with a stable PoCR ~ 0.9 and mean(${\mathrm{HR}}_{\mathrm{Post}}/{\mathrm{HR}}_{\mathrm{Pre}}$) ~ 1. This indicates that slower RT events were not necessarily incorrect. This is an interesting observation and could be due to RT not being a judgment criterion for participants.

## 5. Discussion

In Table 4, we compare this work with the previous monitoring systems [8,49,50,51,52] based on different cognition models, sensor inputs, algorithms, and accuracy achieved. The EEG and ECG have been used most frequently to evaluate physiological signals for cognitive monitoring; however, both require contact electrodes. Our work is based on the simultaneous extraction of respiratory and cardiac features with an over-the-clothes sensor and can achieve relatively high accuracy in addition to deployment convenience and continuous long-term monitoring. Unlike existing RF technologies, this sensor is not affected by ambient motion and does not require the user to be in isolation [32].

The personalized prediction model results in Section 4.1 shows our ability to detect participant’s attentive or relaxed–inattentive states over a 90 s window, when trained over a short duration baseline (3 min for each state). This high-resolution detection can allow for monitoring changes in user’s attentiveness over time. Moreover, our results indicate superior performance of respiratory features for vigilance-based attentive state classification. The nonintrusive, low-cost feature of this sensing technology allows exploration of respiratory signal features with ease for other purposes, unlike a chest belt, which requires sufficient tension, and a nasal flow meter, which is highly intrusive.

**Figure 10:**
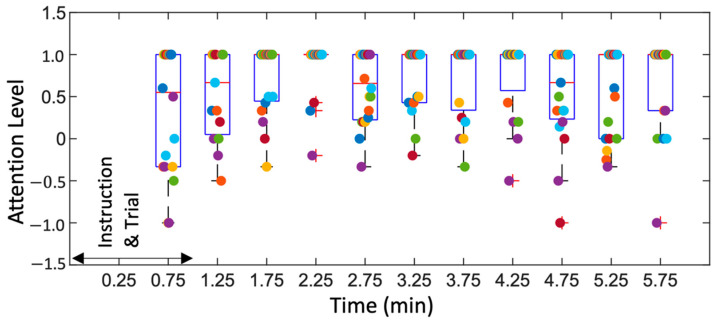
Box plot showing attention levels of participants as the routine progressed. Each participant is indicated by a scatter point in the 30 s time bin.

We also explored change in sustained attention over the test duration, as shown in Figure 10. Here, attention level is defined as a relative ratio of (C − W − M)/(C + W + M), where C, W, and M are correct, wrong, and missed events, respectively. The box plot in Figure 10 show distribution of attention level across participants as time progressed, with each scatter point showing individual participant value. We can observe a slightly lower median value and higher inter-quartile range (IQR) during the initial trial phase and after ~4 min. The trend is reasonable since it took initial 1–2 min for participants to learn the game and then they got drowsy or tired as the game continued for a longer period, based on the reviews noted earlier. This definition of attention has limited scope as it does not include RT, which is shown to be related to attention and fatigue in earlier work [53].

*The studies limitations are as follows*. A major limitation of this study regards the ground truth of attentive and relaxed inattentive states. While we have used an established vigilance-based attention task, multiple participants reported feeling drowsy towards the end due to the monotonous nature. Attention can be interpreted differently as short or sustained attention, and at times may only involve thinking without a quick RT requirement. All these cases may induce different features and may not be generalizable by this model. Further, a relaxation inducing routine is likely dominated by the baseline participant’s feeling of being stressed, happy, or any other emotion that can impact heartbeat and respiratory features differently. This is indicated by the poor leave one subject out performance in this work. In earlier work [48], the baseline variation for one user was studied over multiple weeks, which showed more variation in day-to-day versus each emotion on the same day. Hence, this is an important limiting factor in the attention research domain. Another limitation is the small study duration. This could lead the model to learn short temporal similarity instead of being generalized to minor variability in relaxation or attention levels. It is important to train with optimized features over longer duration and observe performance variation over time for same individual. Lastly, while sensor performance has been established in previous works, it needs to be validated over more age, BMI, and health condition diversity.

*The following future work should be carried out.* The future research efforts should bridge the gap between emotion and attention monitoring by utilizing both HRV and RWV features from the NCS sensor which can also be integrated into the furniture [54] without the participant being aware of their being monitored. This covert sensing will reduce the distraction and nervousness of the participant and, thus, decrease the systematic bias. Furthermore, our sensor can offer critical information for studying RSA, voluntary respiration manipulation, and their effect on cardiovascular [55] and skin conductance changes [56]. This area of research has also been associated with beneficial effects on mental and physical health [57]. Thus, we believe that our sensor hardware and classification algorithms have multi-fold benefits and are valuable in the context of comprehensive healthcare by offering comfortable continuous vital-sign information.

## 6. Conclusions

In this paper, we have demonstrated the use of a noninvasive wearable sensor setup for detecting the relaxed–inattentive vs attentive state of a user. This can pave the way for large-scale future studies, that can potentially mitigate risk factors for life conditions, such as driving, as well as daily cognitive monitoring of elderly patients with dementia. We showed strong reliability of the NCS sensor for cardiac and respiratory variability feature extraction compared to the standard reference ECG and chest belts. Our results indicate a major contribution from the respiratory features for attention detection. To the best of our knowledge, this is the first work using noninvasive respiratory sensing for attention and relaxation-inattention classification from accurate estimates of respiratory features, such as II and EI.

## Figures and Tables

**Table 1:** HRV and RWV classification features.

Signal	Derived-Features
$\mathrm{Heart}\;\left(\mathrm{HRV}\right)$	${\mathrm{HR}}^{*},{\mathrm{IBI}}^{*†},\mathrm{pIBI}50,\mathrm{LF},\mathrm{HF},\mathrm{LF}/\mathrm{HF}$
$\mathrm{Respiration}\;\left(\mathrm{RWV}\right)$	II*†,EI*†,IRI*†,RR*†, IV*†,EIII*†,[SDiy]*†η

Here, $x^{*}$ is $\mathrm{mean}\left(x\right)$, $x^{†}$ is $\mathrm{std}\left(x\right)$, $x^{\eta }\;\mathrm{is}\;\mathrm{mean}\;\left(x\right)/\mathrm{std}\left(x\right)\;,y\;\mathrm{is}\;\left\{\mathrm{II},\;\mathrm{EI},\;\mathrm{IRI},\;\mathrm{IV}\right\},\;\mathrm{and}\;i=\left\{1,2\right\}$.

**Table 2:** Classification algorithm comparison.

Algorithm	CV Accuracy (%)		Sensitivity (%)		Specificity (%)	
	NCS	BIOPAC	NCS	BIOPAC	NCS	BIOPAC
SVM	94.8	94.2	92.0	92.2	96.7	95.5
QDA	91.2	88.4	82.2	75.0	97.4	97.6
Boosted Tree	97.6	96.8	96.8	94.5	98.1	98.4
Bagged Tree	96.4	96.9	95.2	95.2	97.2	98.1
kNN	98.2	98.5	97.5	97.8	98.6	99.0

**Table 3:** Personalized prediction model performance across subjects.

Subject ID	Test Accuracy (%)		Sensitivity (%)		Specificity (%)	
	NCS	BIOPAC	NCS	BIOPAC	NCS	BIOPAC
1	100	100	100	100	100	100
2	100	85.7	100	100	100	83.3
3	33.3	83.3	100	100	20	80
4	100	100	100	100	100	100
5	100	100	100	100	100	100
6	100	100	100	100	100	100
7	21.4	21.4	100	100	8.3	8.3
8	100	85.7	100	0	100	100
9	71.4	100	100	100	66.7	100
10	85.7	85.7	0	0	100	100
11	100	100	100	100	100	100
12	66.7	66.7	100	100	0	0
13	57.1	57.1	100	100	50	50
14	100	35.7	100	100	100	25
15	57.1	28.6	0	0	66.7	33.3
16	71.4	64.3	0	0	83.3	75
17	100	100	100	100	100	100
18	100	85.7	100	0	100	100
19	100	100	100	100	100	100
20	100	100	100	100	100	100
Mean	83.2	80.0	85.0	75.0	79.8	77.8

**Table 4:** Comparison of this work on attention and cognition monitoring with previous methods.

Reference	Cognition Model	Sensor Input	Algorithm	Accuracy (%)	Other
Belle 2012 [49]	Attention	ECG	Random forest	77.0	{Se, Sp}: {66.7, 87.2} %
		EEG	Random forest	85.7	{Se, Sp}: {79.7, 91.7} %
Stancin 2021 [50]	Drowsiness	EEG	XGBoost	59.4	Pr: 59.0%
Barua 2019 [51]	Driver sleepiness	EEG, EOG, Contextual	SVM	93.0	{Se, Sp}: {94.0, 92.0} %
Monkaresi 2017 [8]	Engagement	Video based facial expressions and HR	Naïve Bayes	-	ROC AUC: 75.8%
Patel 2011 [52]	Driver fatigue	ECG HRV	Neural network	90.0	
This work	Attention	NCS HRV and RWV	kNN	83.2	{Se, Sp}: {85.0, 79.8} %

Abbreviations are as follows: AUC, area under the curve; Se, sensitivity; Sp, specificity; Pr, precision.

## Data Availability

Data presented in this study are available on request from the corresponding author for privacy purposes.
